# Influenza A Virus–Host Specificity: An Ongoing Cross-Talk Between Viral and Host Factors

**DOI:** 10.3389/fmicb.2021.777885

**Published:** 2021-11-05

**Authors:** Miaomiao Zhang, Mingbin Liu, Shimeng Bai, Chen Zhao, Zejun Li, Jianqing Xu, Xiaoyan Zhang

**Affiliations:** ^1^Scientific Research Center, Shanghai Public Health Clinical Center & Institutes of Biomedical Sciences, Key Laboratory of Medical Molecular Virology of Ministry of Education/Health, Shanghai Medical College, Fudan University, Shanghai, China; ^2^Shanghai Veterinary Research Institute, Chinese Academic of Agricultural Sciences & Animal Influenza Virus Evolution and Pathogenesis Innovation Team of the Agricultural Science and Technology Innovation Team, Shanghai, China

**Keywords:** influenza A virus, host specificity, pathogenesis, host factors, PB2

## Abstract

One big threat from influenza A viruses (IAVs) is that novel viruses emerge from mutation alongside reassortment. Some of them have gained the capability to transmit into human from the avian reservoir. Understanding the molecular events and the involved factors in breaking the cross-species barrier holds important implication for the surveillance and prevention of potential influenza outbreaks. In this review, we summarize recent progresses, including several ground-breaking findings, in how the interaction between host and viral factors, exemplified by the PB2 subunit of the influenza virus RNA polymerase co-opting host ANP32 protein to facilitate transcription and replication of the viral genome, shapes the evolution of IAVs from host specificity to cross-species infection.

## Introduction

Influenza A virus (IAV) is a major pathogen that greatly impacts human health and the poultry industry with a broad host tropism, capable of infecting various hosts, including wildfowl, swine, bat, and humans being. IAV is a negative-stranded RNA virus comprising eight segments. Two major surface proteins of IAV are hemagglutinin (HA) and neuraminidase (NA), which are used for classifying IAV subtypes based on sequence similarity. Up to date, 18 HA and 11 NA subtypes have been discovered, with H17, H18, N10, and N11 being only found in bats (Tong et al., 2013). Besides HA and NA, the IAV genome encodes at least 16 additional viral proteins, among which PB2, PB1, PA, HA, NP, NA, M1, M2, NS1, and NS2 proteins have been extensively characterized with relatively clear function while PB2-S1, PB1-F2, PB1-N40, PA-X, PA-N155, PA-N182, M42, and NS3 protein represent more recently discovered proteins (Vasin et al., 2014) and their functions remain largely unknown.

Birds are the major, if not the only, natural reservoir of IAV. Avian influenza viruses (AIV) can be divided into low-pathogenic (LPAIV) and highly pathogenic (HPAIV) strains, as determined by the presence of different types of the proteolytic cleavage site in HA (Horimoto and Kawaoka, 2005). The genetic diversity of IAVs is driven by an error-prone replication machinery, in addition to genetic reassortment events where viruses co-infecting the same cell exchange segments. As a result, a cross-species spillover can occasionally occur as an AIV mutant acquires the ability to transmit into aquatic (dolphins, seals, whales) or terrestrial mammals (pigs, horses, mink). Recent reports on human infection of AIV can be traced back to the year of 1997, when the contraction of H5N1 AIV killed 6 people in Hong Kong (Wong and Yuen, 2006). In 2003, an H7N7 AIV outbreak was reported in Netherlands, infecting 84 people with one death (Wong and Yuen, 2006). The threats of transmission of AIV to humans appear to continue growing: local outbreaks of highly pathogenic H5N1 AIV occurred in many Asia countries until 2004 (Stegeman et al., 2004); a total of 1,568 human infection H7N9 AIV has been reported, responsible for at least 617 deaths; the human spillover of H10N8 and H5N6 AIV were also observed (Chen et al., 2014; Yang et al., 2015). These indicate that, although breaking the cross-species barrier is difficult, there is always a likelihood of it happening with an expanding reservoir of AIV. Thus, exploring the molecular mechanisms underlying the host specificity of IAV and the events leading to their breach is an important step toward improving the preparedness of future influenza pandemics by instructing the development of enhanced surveillance of potential risk of avian-to-human transmission; identifying hidden virus–host interfaces as new targets for antivirals. This review will summarize recent advances that start to unfold the complexity of species-specific virus–host interactions underpinning the host range of IAVs.

## Viral Determinants of IAV Host Specificity

A clear announcement of host specificity of IAV is made because avian viruses generally show poor replication in mammalian cells, and vice versa. The host restriction of IAV has been linked to the difference between host of factors governing viral entry and multiple intracellular steps required for viral replication. Consequently, only AIVs with adaptive mutations, which account for a small portion of the virus pool that emerged from the natural reservoir, gain the ability to infect mammalian cells. These mutations are discussed in details per involved viral proteins.

### The Viral HA Protein

The entry of IAV into host cells, representing the first species barrier to overcome, is mediated by viral surface hemagglutinin (HA) glycoprotein. Although all HA proteins have a general cell receptor in sialic acids (SA), a diverse family of sugar units terminally attached to glycans decorating surface glycoproteins and glycolipids, they show an origin-dependent preference for sialic acid-galactose linkage. HAs from human influenza viruses preferably recognize α-2,6 linked SA (α-2,6 receptor) while those of avian origin showed a higher binding affinity with α-2,3-linked SA (α-2,3 receptor; Matrosovich et al., 1997, 2000). The correlation of this recognition pattern with SA distribution in human tissues explains why avian IAV normally cannot infect humans. In humans, α-2,6 receptors are mainly expressed on cells of the ciliated epithelium along the upper respiratory tract (URT), while α-2,3 receptors are primarily found in the lower respiratory tract (LRT; Shinya et al., 2006). In contrast, pig trachea presents both α-2,3 and α-2,6 receptors and is thus susceptible to infection of both human and avian virus, making pig a mixing vessel where new viruses can be generated through reassortment from parental virus of different origins.

Extensive structural studies have revealed the modes of interactions between HA and their corresponding SA receptor (Long et al., 2019). A wider receptor binding site (RBS) has been posited to form the basis of human-adapted HA accommodating α-2,6 SA linkage, which appears bulkier than the α-2,3 SA linkage. Several adaptive mutations in RBS of avian HAs have been shown to cause a switch of binding specificity, exemplified by E190D/G225D and A138S in H1 HA and Q226L/G228S in the HAs of H2 and H3 subtypes (Shi et al., 2014). These mutations increase binding affinity to the human receptor while reducing the binding to the avian receptor. The HA proteins of recently emerged H7N9 AIV that showed high pathogenicity in human infections appear to acquire some ability to bind to human receptor despite maintaining the preference for the avian receptor (Xu et al., 2013). This change was particularly exhibited by the Anhui-H7N9 virus, which harbors four mutations in RBS, namely S138A, G186V, T221P, and Q226L, accounting for the shift of receptor binding preference (Shi et al., 2013). As for the H5N1 virus, those causing human infections retain preference for avian receptor but such specificity can be skewed to human side by HA mutations in position 226 and 228, which are also required for airborne transmissibility (Stevens et al., 2008). Overall, typical AIV strains have not yet shown acquisition of transmissibility among humans until now. Despite the odds, the possibility of AIV breaking such a barrier cannot be ruled out, especially considering that multiple types of HA can attain airborne transmission ability in mammalian model systems (Kuchipudi et al., 2021).

### The Viral NA Protein

As another major viral surface protein alongside HA, NA possesses sialidase activity and is responsible for cleaving SA from the cell surface to facilitate the release of the progeny virus particle (Schrauwen et al., 2014). Strong evidence supporting the adaptation of NA to host came from the short-stalk NA favored by poultry IAV, which is speculated to match short length of glycan linked to SA receptor (Li et al., 2010). However, this type of NA does not support airborne transmission in ferrets, likely due to ineffective cleavage with longer glycan and the resultant virus clumping. Thus, it has been proposed that a functional balance between the SA binding of HA and NA sialidase activity is a critical element involved in the host fitness of IAV (Lakdawala et al., 2015). Interestingly, a second HA binding site was found on some NAs, contributing to their hemadsorption (Hd) activity. Such Hd site was originally thought to regulate the catalytic activity of NA positively, but the recent characterization of NA protein from the H7N9 virus indicated that its existence might allow enhanced receptor binding, particularly to human-like α2,6-linked sialic acid (Benton et al., 2017). It remains to be determined whether the Hd site contributes to the ability of the H7N9 virus to infect humans infection.

### The Viral Polymerase Proteins

The transcription and replication of the viral genome, carried out by the viral RNA polymerase, are central to viral growth and consequently become a critical barrier to overcome during the adaptation of IAV to a new host. The tripartite RNA-dependent RNA polymerase (RdRp) of IAV consists of polymerase protein basic 1 (PB1), polymerase protein basic 2 (PB2), and polymerase acidic protein (PA). Inside the virus, all the eight viral RNA (vRNA) segments are coated by nucleoprotein (NP) with RdRp binding to the conserved 5′ and 3′ ends to form viral ribonucleoprotein (vRNP) complexes (Nilsson et al., 2017). Once transported into the nucleus in infected cells, RdRp first transcribes vRNA into mRNA for viral protein production, a process initiated by a cap snatching mechanism wherein a nucleotide sequence of 10–20nt length is cleaved from the 5′ end of host mRNAs by a sequential action of PB2 and PA and subsequently serve as the primer for viral mRNA synthesis (Dias et al., 2009; Yuan et al., 2009). The replication of the viral genome ensues after accumulation and nuclear transport of newly synthesized RdRp proteins. In contrast to transcription, the RdRp-mediated replication is primer independent and involves cRNA as an intermediate product. Additionally, NP is required for replication while dispensable for transcription. Recent studies also suggest that the catalytic activity of RdRp in vRNA replication requires dimer formation between an RNA-bound RdRp and free RdRp, which acts as a relay station to synthesize vRNA from cRNA (Nilsson et al., 2017). The central domain of RdRp is composed of PB1, the C-terminal domain of PA, the N-terminal one-third of PB2 encompassing the N terminus, the lid domain, and the N1 and N2 linkers (Nilsson et al., 2017).

The host adaptation of IAV RdRp is best displayed on PB2, which is responsible for cap-binding and participates in NP binding *via* regions in its N- and C-terminals. Several mutations in PB2 have been identified to be important for promoting adaptation of avian IAV to mammalian, epitomized by Glu-to-Lys substitution at position 627 (E627K) and Asp-to-Asn substitution at position 701 (D701N; Gabriel and Fodor, 2014). Subbarao et al. first reported the identity of amino acid at position 627 as a dividing line between avian and human influenza virus with respective E and K dominance (Subbarao et al., 1993). Importantly, a single change of E to K at position 627 was sufficient for restoring the ability of a PB2 single gene reassortant to replicate effectively in canine (MDCK) cells (Subbarao et al., 1993). Further analysis revealed that PB2 E627K mutation could remarkably raise the RdRp activity in mammalian but not avian cells, leading to an increased virulence in mammalian hosts’ pathogenicity (Subbarao et al., 1993; Hatta et al., 2001; Gabriel et al., 2005). Indeed, 627K is commonly shared by a wide spectrum of human pathogenic IAVs, including H5N1, H7N9, H10N8, and H5N6 subtypes (Gao et al., 2013; Garcia-Sastre and Schmolke, 2014; Yang et al., 2015). D701N as a mammalian-like signature was first identified by Li et al. through analyzing the genetic determinant underlying the ability of a duck H5N1 IAV to replicate in mice (Li et al., 2005). More recently, some H9N2 viruses isolated from minks were reported to contain the PB2 701N mutation and consequently showed enhanced virulence in mice compared to those without such mutation, supporting the potential of mink as a mixing vessel or intermediate host for H9N2 virus and possibly other IAVs (Xue et al., 2018).

Interestingly, other PB2 mutations, though less frequent than 627K or 701N, can also promote human adaption. For example, the 2009 pandemic H1N1 virus harbors neither PB2 627K nor PB2 701N signatures (Mehle and Doudna, 2009). Instead, its mammalian adaptation was supported by PB2 G590S/G591R polymorphisms, which also conveyed enhanced RdRp activity in human cells (Mehle and Doudna, 2009). PB2-Q591K and K526R are two other mutations shown in H9N2 virus and H7N9/H5N1 viruses capable of conferring effective replication in mammalian cells in the absence of 627K and 701N (Song et al., 2014; Wang et al., 2016). The combinatorial effects of different adaptive mutations have also been studied. H7N9 virus bearing both 627K and 526R replicates more efficiently in mammalian cells than those carrying the individual mutation, consistent with greater mortality in mice (Song et al., 2014). Mechanistically, the combination of 526R with 627K appeared to optimize the interaction between PB2 and nuclear export protein (NEP), thereby promoting higher polymerase activity. A similar synergistic effect between PB2-526R and 701N was also documented. In contrast, the co-existence of PB2 E627K and D701N mutations has not yet been detected in naturally occurring virus isolates, suggesting potential functional redundancy of the two mutations (Steel et al., 2009; Gabriel and Fodor, 2014).

Adaptive mutations of other components of IAV replication machinery were also identified, despite being less frequent than what was seen with PB2. A divergency at the position of PB1 protein of human influenza A/H1N1 viruses was recently identified: This position was predominantly occupied by the avian-associated serine residue before switching to mammalian-associated glycine residue near the onset of the 2009 pandemic (Lin et al., 2019). Subsequent studies showed that PB1-216G was a lower RdRp fidelity variant than PB1-216S, thereby allowing the H1N1 virus to yield adaptive mutations at higher rates and promoting viral epidemiological fitness (Lin et al., 2019). Multiple studies revealed the role of PA in the human adaptation of AIV. It was found that, in avian H9N2 background, a single replacement of PA segment from 2009 pandemic H1N1 virus resulted in enhanced viral polymerase activity, leading to a higher pathogenicity in infected mice (Sun et al., 2011). A systematic comparison of a panel of reassortant viruses between a duck-derived H5N1 and a highly transmissible human-infective H1N1 virus showed that PA and nonstructural gene are the major H1N1 determinants of droplet-mediated transmission between guinea pigs (Zhang et al., 2013). A couple of human adaptive PA mutations were identified, including E349G and PA 97I, with a positive impact on viral polymerase activity in mammalian cells (Song et al., 2009). Evidence supports the concerted action between mutations of different RdRp subunits and NP protein in enhancing human adaptation. Gabriel et al. reported that mutations in PB2, PA, and NP, namely PB2 701N and 714R, PA 615N, and NP 319K, cooperatively enhance AIV SC35 (H7N7) polymerase activity and consequently enable an effective replication in mammalian cells (Gabriel et al., 2005). The coupling between PB2 D701N and NP D319K mutation was confirmed in other studies (Gabriel et al., 2011). The molecular mechanism(s) underpinning synergy between these mutations in enhancing mammalian fitness of AIV has yet to be fully delineated, though some studies, as described below, suggested the involvement of importin-α.

### NS1A and Other Viral Proteins

The interferon (IFN)-centered innate immune response represents the first-line defense against viral infection. Accordingly, IAV develops several viral countermeasures epitomized by nonstructural protein 1 (NS1). NS1 protein is produced by the co-linear transcript of RNA segment 8, which also encodes NS2 (NEP) through alternative splicing (Liu et al., 1997). NS1A comprises an RNA binding domain (RBD) and an effector domain (ED); both domains contribute to the ability of NS1A to use multiple rather than single tactics to evade the IFN antiviral response (Yin et al., 2007; Bornholdt and Prasad, 2008). Two main mechanisms have been proposed for the IFN countering the action of NS1A, namely blocking the activation of RIG-I, the major pattern recognition receptor responsible for sensing the incoming IAV genome, and inhibiting the activity of CPSF30 (Cleavage and polyadenylation specificity factor 30kDa subunit) from suppressing the maturation and consequently the nuclear export of IFN mRNA (Cho et al., 2020; Rosário-Ferreira et al., 2020). The RNA-binding of NS1, once thought to sequester dsRNA produced by viral replication from host dsRNA sensor, was shown to primarily function as a competitor of 2′-5′-oligoadenylate synthetase (OAS) to suppress the RNaseL antiviral pathway (Min and Krug, 2006). NS1A is also engaged in interactions with various host factors without known roles in innate immune response, implicating additional regulatory roles in other aspects of the viral cycle (Rosário-Ferreira et al., 2020). The wide spectrum of host protein interactions by NS1A has been proposed as a result of the ready-for-exploitation nature of its ED (Cho et al., 2020) and/or a double-stranded RNA platform that strengthens the weak interactions NS1A with certain host factors (Chen et al., 2020).

Several naturally occurring mutations of NS1 have been linked to enhanced viral replication and pathogenicity, including D92E, P42S, and deletion of 80-84 residues (del80-84). As expected, these mutations were often associated with increased virus resistance to interferon (Seo et al., 2002). It is a bit of surprise that the CPSF30 binding site of NS1A is not conserved across IAVs: Although the majority of NS1A proteins bind to CPSF30, those from pandemic H1N1 2009 virus and human-infective H5N1 1997 virus cannot (Long et al., 2019), which leads to a hypothesis that gain or loss of CPSF binding of NS1A may be a viral means to balance between viral replication and interferon antagonism on the road toward mammalian adaptation. Sequence analyses also pinpoint several amino changes that could associate with the adaptation of AIV to mammalian host, such as the alteration of last amino acids from ESEV to RSKV (Zielecki et al., 2010), and the D125G substitution introducing an extra splice site into the NS gene (Selman et al., 2012). However, whether and how these changes may influence the host range of AIV remained to be determined. Overall, a link between NS1 mutations and cross-species transmission of AIVs is still elusive.

Apart from NS1A, the human adaptation of AIV may be facilitated by mutating viral accessory proteins that were recently discovered, such as PB1-F2 and PA-X. PB1-F2 is an 87-amino acid small protein produced from PB1 encoding sequence through +1 frameshift and has been shown to antagonize antiviral innate immunity possibly *via* interaction with components of the RIG-I/MAVS system, particularly the mitochondrial antiviral signaling (MAVS) protein (Mazur et al., 2008). Interaction between PB1-F2 and PB1 has also been documented and is in line with the enhancing effect of PB1-F2 on the RdRp activity (Mazur et al., 2008). The role of PB1-F2 in the viral pathogenesis was highlighted by the finding that a single amino acid substitution in PB1-F2, namely N66S, is associated with increased virulence of the 1997 human H5N1 virus is also conserved in the 1918 pandemic virus (Conenello et al., 2007). However, the ORF of PB1-F2 is often truncated or lost in swine or human IAVs, raising the speculation that PB1-F2 is not essential for IAV mammalian adaptation or a compensatory mechanism has been acquired (Long et al., 2019).

PA-X is the product of +1 ribosomal frameshifting during translation of PA protein, retaining the 191-aa long N-terminal endonuclease domain while acquiring a unique C-terminal region known as X-ORF, commonly encoding 61 aa (Jagger et al., 2012). PA-X is known to facilitate viral growth by coordinating the virus-mediated shutoff of host gene expression (Gaucherand et al., 2019). Interestingly, some IAV strains possess early stop codon in the X-ORF, resulting in a truncated PA-X protein 41 aa in length (Sun et al., 2020). Avian, equine, and human seasonal H3N2 and H1N1 influenza viruses generally encode full-length X-ORF while truncated X-ORF is characteristic of the 2009 pandemic H1N1, swine and canine viruses (Sun et al., 2020). In terms of swine influenza viruses (SIVs), the majority of early isolates were found to express a full-length PA-X until 1985, from when isolates featuring truncated PA-X gradually increased to become the dominant species, implicating a selection favoring truncated PA-X in pigs (Xu et al., 2016). Indeed, Xu et al. demonstrated that, in the background of a “triple-reassortment” H1N2 SIV, mutating X-ORF to express a full-length PA-X resulted in attenuated viral replication and transmission in pigs (Xu et al., 2017). By contrast, an investigation based on the 2009 pandemic H1N1 revealed that full-length PA-X protein supported increased virulence in mice relative to the truncated form (Gao et al., 2015). Thus, PA-X is a likely contributing factor to the host specificity of IAV. The notion was further strengthened by the finding that single human-specific amino acid substitutions in PA-X, particularly the R195K mutation, might underpin the animal-to-human jump of H7N9, H5N6, and H1N1/2009 viruses (Sun et al., 2020).

## Intracellular Host Factors Underlying the Host Specificity of IAV: Importin-α and ANP32A Take the Lead

A major recent advance in understanding the host specificity of IAV is identifying the related intracellular host factors, best represented by importin-α and ANP32 proteins.

### Importin-α

In uninfected cells, importin-α functions as an adaptor protein involved in the nuclear transportation of host proteins possessing nuclear localization signal (NLS; Stewart, 2007). IAV-infected cells are usurped by the virus to transport incoming vRNPs into the nucleus and are also responsible for nuclear importation of newly synthesized PB2 and NP proteins following primary transcription, which is a requisite for sustained viral replication (Stewart, 2007). Avian and human IAVs appear to engage different importin-α isoforms for effective viral replication as revealed using importin-α-silenced cells and importin-α-knockout mice: Avian virus depends on importin-α3, whereas human virus displays a switch to importin-α7 dependency; the 2009 H1N1 pandemic IAV instead showed a dual dependency of both importin-α3 and importin-α7, suggesting ongoing viral adaptation (Gabriel et al., 2011; Pumroy et al., 2015). Consistent with the viral replication data, avian- and human-like PB2 were found to bind preferentially to importin-α3 and importin-α7, respectively (Gabriel et al., 2011). Further evidence that differential importin-α recognition is a mechanism of IAV host specificity came from binding studies that D701N mutation enhances the interaction between PB2 and importin-α7, so does D319K for NP (Gabriel et al., 2011). Given the proximity of 701 to the NLS of PB2, it has been speculated that the D701N mutation might induce a conformation change that facilitates a stronger binding to importin-α (Tarendeau et al., 2007; Sediri et al., 2015). Interestingly, a most recent study identified a correlation between PB2-D701N/NP-D319K mutations and reduced expression of importin-α3 in the respiratory tract of IAV-infected humans and mice. As importin-α3 mediates the nuclear import of NF-κb and is thus required for induction of various antiviral genes in IAV-infected cells, its downregulation complements the virus’s capability to access importin-α7 in ensuring that virus can gain replicative fitness in the human lung (Gabriel et al., 2008; Thiele et al., 2020).

### ANP32 Proteins

**Figure 1:**
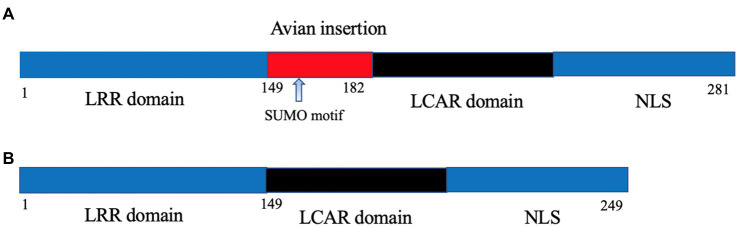
Schematic illustration of chicken **(A)** and human ANP32A **(B)**. The avian-specific 33-amino acid insertion is highlighted in red with the containing SUMO binding motif being indicated by an arrow.

Once in the nucleus, the IAV viral polymerase needs to utilize host machinery for effective transcription and genome replication, and this represents another major barrier that AIV needs to surpass upon avian-mammalian adaptation. The original clue for this barrier came from the observation that the poor activity of avian IAV polymerase in mammalian cells could be rescued by fusion with avian cells to form heterokaryons, which can be explained by the absence of a supportive avian host factor in mammalian cells. Long et al. first discovered this longed-for host factor as chicken ANP32A. Surprisingly, there is a human homolog of chicken ANP32A (chANP32A); the major difference between chicken and human ANP32A is that the former possesses an additional 33 amino acids between the shared leucine-rich repeats and carboxy-terminal low-complexity acidic region domains owing to exon duplication (Long et al., 2016; Figure 1). Importantly, the 33-aa insertion was responsible for the superiority of chicken ANP32A over human ANP32A in supporting the activity of avian PB2-627E viral polymerase in mammalian cells (Long et al., 2016; Domingues and Hale, 2017). In this regard, the PB2 E627K mutation was selected to meet the viral need to co-opt the mammalian ANP32A for effective viral replication.

Subsequent studies further revealed deep connections between ANP32 family proteins and human adaption of IAV polymerase. The activity of ANP32A appears to be regulated by alternative splicing in avian cells, resulting in variants containing essential no (human-like), partial, or full 33aa-insertion (Baker et al., 2018). More interestingly, the usage of ANP32A splicing sites varies across different bird species, and some species predominantly express human-like ANP32A, most pronounced in swan goose (>70%) and ostrich (close to 100%; Domingues et al., 2019). It is thus proposed that the differential ANP32A splicing inherent to natural avian hosts may provide a pre-adaptation mechanism that shapes the evolution of PB2 to facilitate cross-species transmission (Domingues et al., 2019). A recent finding further strengthened the view of ANP32A as a facilitator of IAV evolution. Swine ANP32A is capable of supporting avian-type IAV polymerase activity, although to a less extent as compared with avian ANP32A, consistent with the pig as an important intermediate host for zoonotic IAVs (Zhang et al., 2020a). This unique feature of swine ANP32A was attributed to 106V, which exhibits positive epistasis with 156S and has not been found in other studied vertebrate species (Zhang et al., 2020a). Recent studies also revealed another member of ANP32 family, ANP32B, in regulating IAV polymerase activity. This revelation was made using human ANP32A and ANP32B single- and double-knockout cells as only in the latter cells was the viral polymerase activity abrogated (Zhang et al., 2019). The analysis also indicated that human ANP32B is a more potent pro-viral factor than human ANP32A. In contrast, the viral polymerase supporting activity was lost in chicken ANP32B, and further dissection pinpointed residue 129 and 130 as the critical determinant of ANP32B activity: Avian ANP32B possesses isoleucine and asparagine at the two positions, whereas in human ANP32A and ANP32B, as well as avian ANP32A, they are filled with asparagine and aspartate (Zhang et al., 2019).

The precise mechanism by which ANP32 regulates the activity of IAV RNA polymerase remains unclear. ANP32A or ANP32B was associated with the trimeric viral polymerase in infected cells or when they were simultaneously expressed by co-transfection (Zhang et al., 2019). The failure of ANP32A to bind to free PB2 in co-expressed cells indicated that such association requires the formation of a viral polymerase complex. On the other hand, *in vitro* binding assay employing purified protein revealed a direct interaction between ANP32A and the 627 domain of PB2. However, the binding of human or chicken ANP32A to viral polymerase appears to be independent of PB2-627 identity (Domingues and Hale, 2017). Following the finding that chicken ANP32A binds to PB2 stronger than human ANP32A, Domingues et al. discovered a sumo interaction motif (SIM) in the first four residues of the avian-specific 33-aa insertion and suggested it might account for increased binding of chicken ANP32A to PB2 (Domingues and Hale, 2017). However, a natural splicing variant of chicken ANP32A with SIM deletion can support viral polymerase activity, though less effective than that with a complete 33-aa insertion (Domingues and Hale, 2017). Recently, Baker et al. provided experimental evidences linking ANP32A to a specific form of the viral polymerase (Baker et al., 2018). The binding of ANP32A to viral polymerase in co-transfected cells was enhanced in the presence of viral genomic RNA. It was known that the low activity of PB2 627E viral polymerase can be trans-complemented by mutations at positions 3 and 5 of 3′ vRNA promoter. When assayed on reporter bearing such mutant vRNA promoter, chANP32A showed a little enhancing effect, implying the same or similar mechanism shared by a mutant promoter and chANP32A (Baker et al., 2018). These results are consistent with the hypothesis that ANP32A facilitates the assembly of either cRNP, vRNP, or both.

Structural approaches have also searched the molecular basis for recognition of ANP32A by PB2. Using a combination of NMR and quantitative ensemble analysis, Camacho-Zarco et al. conducted a comparative analysis of complex formed between human ANP32A and human-adapted PB2-627-NLS domain (K627 form) versus that comprising avian ANP32A and avian adapted PB2-627-NLS domain (Camacho-Zarco et al., 2020). Although both complexes were highly dynamic, they were found to display two different modes of interaction: Human ANP32A utilizes a track of positively charged residues including K627 to maximize interaction with highly acidic IDD domain (alternative name of LCAR to highlight its low complexity); with such positive charged surface being interrupted by E627, avian ANP32A can compensate this loss by broadening the sampling of IDD domain to exploit more expanded interaction surface, particularly involving a hexapeptide motif present in the avian-specific 33-aa insertion (Camacho-Zarco et al., 2020). Despite providing the first structural insight into host-specific ANP32A-PB2 interaction, the study from Camacho-Zarco et al. could not interpret the previous observation that chicken ANP32A binds to PB2 more strongly than its human ortholog. Instead, it would predict a higher binding affinity between human ANP32A and 627K type PB2. More recently, Carrique et al. reported cryo-EM structures of influenza C virus polymerase (FluPolC) in complex with human or avian ANP32A (Carrique et al., 2020). In both structures, two FluPolC molecules, one bound to a 47-nt long vRNA and one staying free, formed an asymmetric dimer that ANP32A bridges through its LRR domain (Carrique et al., 2020). The LCAR domain of ANP32A further stabilizes the complex formation in the FluPolC-chicken ANP32A structure with the avian-specific 33-aa insertion directly interacting with the two juxtaposed 627 domains of PB2 (Carrique et al., 2020). The authors thus proposed that ANP32A-FluPol dimer functions as a platform for viral genome replication, where a vRNA-bound FluPol_R_ and a free FluPol_E_ act sequentially in cRNA synthesis and the ensued vRNP assembly (Carrique et al., 2020). However, chicken ANP32A, unlike human ANP32A/B, has limited ability to support the viral polymerase of the influenza B virus (Zhang et al., 2020b), whether such platform is shared by IAV viral polymerase remains to be determined.

## Conclusion

The host specificity of the IAV is underpinned by complex interactions between viral proteins and host factors. This complexity is translated to the requirement of a constellation of mutations for the virus to adapt to a new host. Although recent studies begin to reveal the molecular basis of several human adaptive mutations, exemplified by PB2 627K, many questions remain to be addressed. For example, no clear consensus has been reached regarding the exact mechanism by which ANP32A is co-opted to enhance viral polymerase activity in a species-specific manner. To answer this, we might need a structure of ANP32A in complex with the matched viral polymerase of IAV. It is also unclear when the human adaptive mutations are selected in the human cells after transmission or preexisted in the animal side. In the case of PB2 627K, the presence of human-like ANP32A in some bird species and the ability of swine ANP32B to support a certain degree of PB2 627K viral polymerase activity would suggest the second possibility. Identifying PB1–216G, which lowers the viral polymerase activity, reminds us that changing the intrinsic property of viral polymerase may lead to adaptive mutations.

Developing an effective countermeasure against innate immunity of new host is another essential stepstone for AIV to cross the species barrier. It is recognized that the outstanding ability of chicken cells to support AIV growth is at least partially ascribed to a compromised type I IFN system, i.e., lacking of a functional RIG-I homolog (Barber et al., 2010). In addition, the chicken version of Mx protein, which is a major IFN-induced anti-influenza effector molecule in mammal setting, is not capable of restricting influenza virus replication (Benfield et al., 2008). NS1A is the most likely viral candidate taking the assignment to cope with the challenge of more powerful innate immunity in mammal cells. Elucidating the relationship between the evolution of NS1A and adaptation of AIV to mammalian hosts and the underlying mechanism warrant future investigation.

It should also be noticed that environmental and physiological factors, which we have not discussed here, also play an important role in the host restriction of IAV, particularly the transmissibility, which adds more constraints to inter-species transmission. A critical hidden constraint is recently revealed by an elegant study of Soh et al., demonstrating that the architecture of genome code makes some potential human adaption mutations impossible because they are inaccessible to single nucleotide substitution (Soh et al., 2019). Extension of the comprehensive, active search for potential human adaption mutations to other viral proteins might provide us a map of risk spots on viral proteins that we need to be aware of when evaluating the cross-species transmission potential of avian viruses. Finally, further exploration of mechanisms underlying the host specificity and cross-species infection is expected to help dissect the complicated network of host factors usurped by IAVs, thus providing new targets for developing therapeutics and antiviral drugs.

## Author Contributions

XZ, JX, and ZL contributed to conception and design of the study. MZ, ML, and SB organized the database and performed the statistical analysis. MZ and CZ wrote the manuscript. All authors contributed to manuscript revision and approved the submitted version.

## Funding

This work was supported by the National Natural Science Foundation of China (81771704, 82071788, 31902276), the Shanghai Pujiang Program (19PJ1409100), and the intramural Funding from Shanghai Public Health Clinical Center (to MZ).

## Conflict of Interest

The authors declare that the research was conducted in the absence of any commercial or financial relationships that could be construed as a potential conflict of interest.

## Publisher’s Note

All claims expressed in this article are solely those of the authors and do not necessarily represent those of their affiliated organizations, or those of the publisher, the editors and the reviewers. Any product that may be evaluated in this article, or claim that may be made by its manufacturer, is not guaranteed or endorsed by the publisher.
